# Characterization of external optical crosstalk reduction for SiPM-based scintillation detectors with an optical bandpass filter

**DOI:** 10.1016/j.nima.2024.169101

**Published:** 2024-01-16

**Authors:** Seungeun Lee, Woon-Seng Choong, Joshua William Cates

**Affiliations:** Applied Nuclear Physics Program, Nuclear Science Division, Lawrence Berkeley National Laboratory, 1 Cyclotron Rd, Berkeley, CA, United States

**Keywords:** Silicon photomultipliers, Optical crosstalk, Optical filter, Scintillation detectors

## Abstract

External optical crosstalk remains a significant source of correlated noise in scintillation detectors optically coupled to silicon photomultiplier (SiPM) arrays and can influence achievable performance by limiting the maximum overvoltage at which a detector can be operated. This study evaluated the potential benefits of incorporating an optical bandpass filter, which is absorptive to optical crosstalk and transmissive for scintillation photons, between the scintillator and SiPM array. Several SiPM and bandpass configurations were characterized, including single-element and SiPM arrays with bare, filter-coupled, crystal-coupled, and crystal-filter-coupled interfaces. Coupling the optical filter between a 4 × 4 array of Broadcom AFBR-S4N44P164 M (NUV-MT) SiPMs and a 12 × 12 × 20 mm^3^ LYSO crystal decreased the total number of detected optical photon noise by up to 92%. The impact of the filter on crosstalk probability reduction was also characterized by the single-channel measurements with the same SiPM, suppressing external crosstalk up to 64%. With the filter coupled, SiPMs were operable at very high overvoltage. The results of these studies demonstrate that optical filtering is a promising technique to significantly reduce correlated noise in scintillation detectors employing SiPMs.

## 1. Introduction

The silicon photomultiplier (SiPM) is a solid-state photosensor with single photon resolving capability, making it a highly promising technology for a wide range of scientific and industrial applications [1–3]. Each SiPM channel consists of thousands of microcells, also known as single photon avalanche diodes (SPADs), operated in Geiger mode and connected in parallel to a common output terminal. When a single photon is detected in the SPAD, it triggers the generation of an electron-hole pair, followed by an avalanche process, resulting in a significant amplification of the single photon response. In SiPMs, the amplitude of generated signal is proportional to the number of detected photons, enabling energy-sensitive readout of radiation interactions within, for example, an optically coupled scintillation material. As advancements continue to be made in the photodetection efficiency (PDE), single photon time resolution (SPTR), noise, and robustness, the application of SiPMs, they are increasingly employed in a diverse range of radiation detection fields, including particle physics [4], high-energy physics [5], medical imaging [6,7], nuclear security [8,9], and environmental radiation monitoring [10,11]. SiPMs are also widely used in optical photon detection applications, such as light detection and ranging [12], Cherenkov telescopes [13,14], and optical imaging [15].

**Fig. 1.**
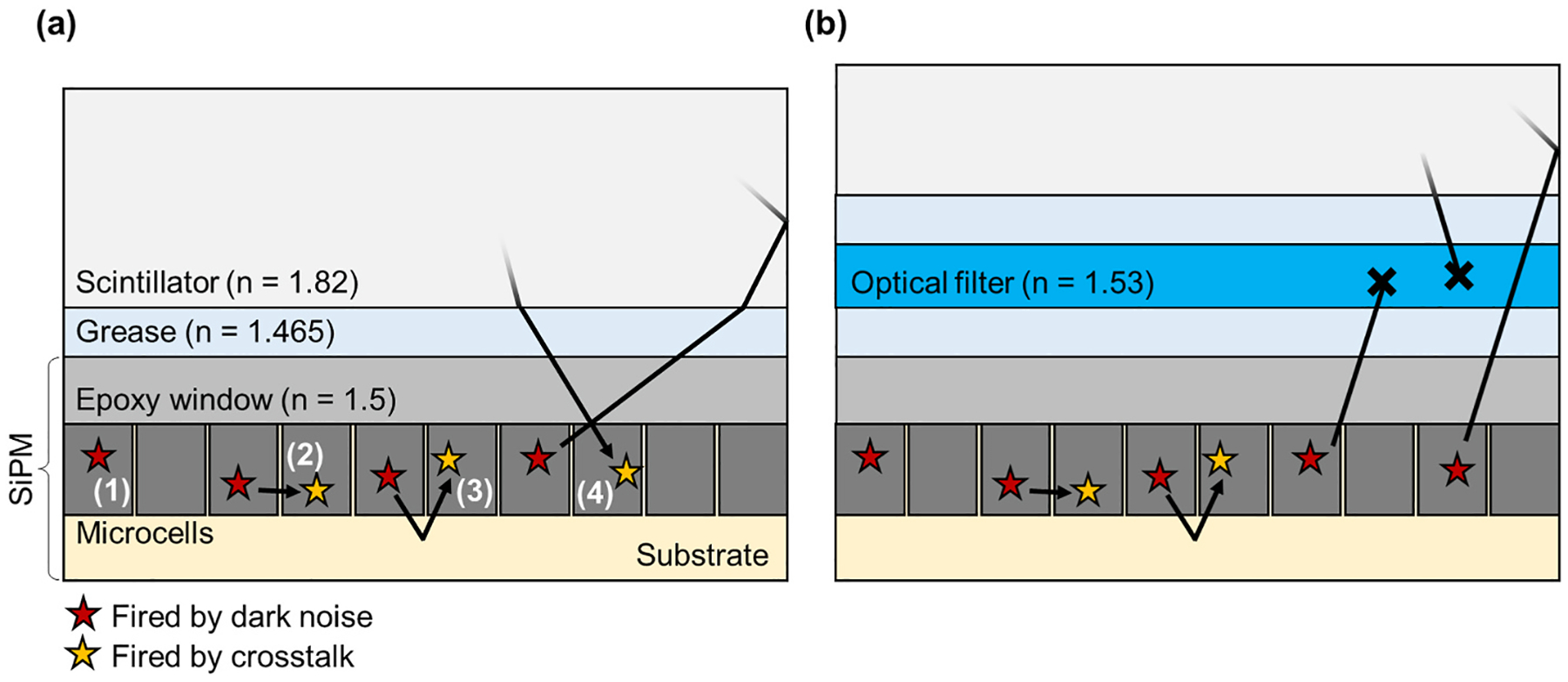
Illustrations of SiPM noise propagation. The events indicated as red and yellow stars correspond to the dark noise and optical crosstalk, respectively, that trigger the avalanche process and the following single photon response. (a) Typical noise types in SiPM coupled with a scintillator include (1) dark noise, (2) direct crosstalk, (3) delayed crosstalk, and (4) external crosstalk. (b) Strategy of external crosstalk suppression using an optical filter.

One drawback of SiPMs is their noise event rate, which is several orders of magnitude higher than vacuum photosensor technologies. Fig. 1(a) summarizes the dominant types of noise generated by SiPM itself [3,16]. “Dark noise” pulses result from thermionic electron emission, which creates single photon equivalent signals in the SiPM. The avalanche also generates optical photons which can subsequently cause neighboring microcells to fire, in a process referred to as “optical crosstalk”. This secondary avalanche is correlated in time with the primary avalanche and, as such, is also referred to as “correlated noise”. A crosstalk photon can trigger secondary avalanches in adjacent microcells by direct detection (direct crosstalk) or after a certain temporal delay by diffusion of electron-hole pairs created outside the active region (delayed crosstalk). A third type is referred to as “external crosstalk” wherein additional microcells trigger from the detection of optical crosstalk photons after propagating through optical media external to the device such as the SiPM’s protection window or scintillation crystal interfaces [17,18]. Crosstalk appears as a pileup of the single photon pulse (mostly direct crosstalk, shortly delayed crosstalk, and external crosstalk), or another photon pulse separated from the primary event. Therefore, these correlated noise signatures contribute to uncertainty in spectroscopy and event timing [3]. Because both dark count rate and crosstalk probability increase with SiPM overvoltage, parameters that improve with increased applied overvoltage (e.g., PDE and SPTR) come at the expense of increased noise. This effect can be especially problematic for SiPM arrays coupled to scintillation crystals, which create transparent return paths for optical crosstalk photons emitted from the SiPMs.

**Fig. 2.**
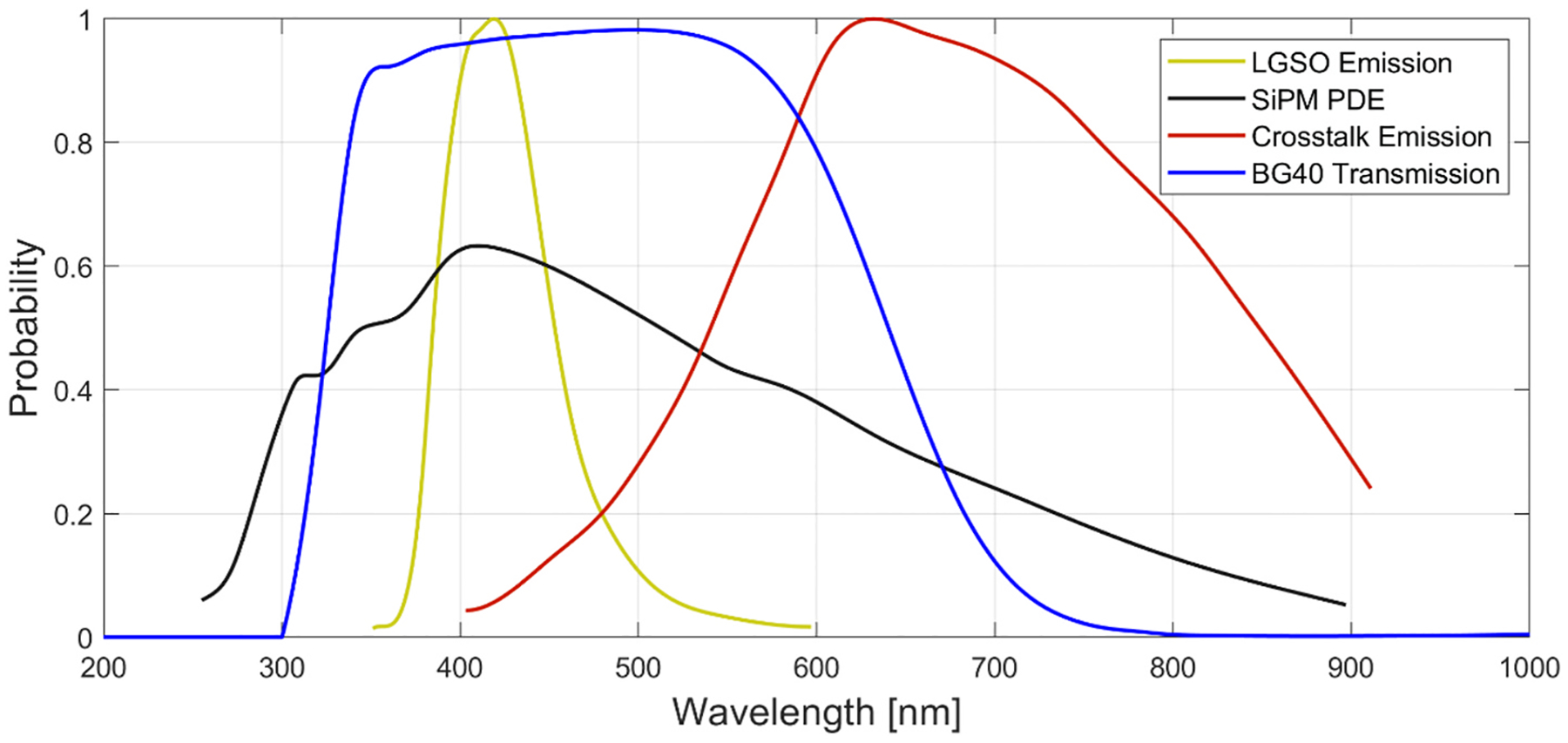
Spectra of LGSO scintillation emission, SiPM photodetection efficiency (PDE), crosstalk photon emission, and BG40 filter transmission. The emission spectra are normalized. The SiPM PDE and crosstalk emission spectra were repurposed from Refs. [18,20], respectively.

Previous works have proposed or demonstrated the potential to significantly reduce external optical crosstalk by employing optical filters [19,20]. Since the emission spectrum for optical crosstalk photons spans a range of approximately 500–900 nm, optical filters that are absorptive in this region (and transparent above and below this window) can be employed to significantly reduce the influence of external crosstalk. What’s more is the crosstalk photon emission spectrum peaks above 600 nm, which is higher than many scintillation crystal emissions spectra. Thus, an optical bandpass could be employed that is transparent to scintillation emissions and absorptive to external optical crosstalk, thereby largely reducing or eliminating this noise contribution from the generated detector signal. As an example, the peak wavelength of lutetium oxyorthosilicate (LSO)-based scintillators typically lies around 420 nm approximately 200 nm below peak wavelength emission for optical crosstalk photons (Fig. 2) [21]. In practice, a non-negligible fraction of crosstalk photons can be detected because of the overlap with the PDE spectrum of SiPM [22]. For this case, a 1-mm thick Schott BG40 filter, for example, can filter out 63% of incident crosstalk photons while maintaining 97% of the useful scintillation photons. Therefore, an optical filter is expected to absorb a large amount of external crosstalk when it is placed between the SiPM and the blue-emitting scintillator (Fig. 1(b)). Previous measurements employing an optical bandpass filter onto bare SiPM arrays exhibited a remarkable reduction in the crosstalk probability of a SiPM array [19]. Given the promise of this technique, the optical bandpass approach should also be evaluated for SiPM-based scintillation detectors, where scintillator volumes with optical reflectors exacerbate the issue of external optical crosstalk, which has been detailed in other works [17]. If successful, this technique could provide a significant reduction in correlated noise rate and potentially enable the operation of scintillator-based detectors with SiPM arrays to operate at room temperature with very high overvoltage, thereby enabling additional benefits that improved PDE, signal-to-noise ratio, and SPTR can provide.

In this work, we investigated the impact of an optical bandpass filter on the noise properties of scintillator-based detectors. We focus on the empirical properties that may be employed in detector simulations (for example via the methods outlined in Refs. [23,24]). Respective contributions of the filter and crystal to the noise properties were characterized by comparing configurations with and without coupling to SiPMs. First, we measured the noise rate and probability of pileup due to the crosstalk of a SiPM array. “SiPM array” indicates the SiPM channels arranged in an array, packaged as a single device by the manufacturer. Thus, the inter-channel effect of the noise was studied comprehensively. An in-depth analysis of crosstalk probability and time delay was performed through a single-channel SiPM experiment, where the inter-channel effect was absent. Lastly, a preliminary investigation of the effect of the filter on achievable coincidence time resolution (CTR) was performed with coincidence measurement between single channel detector pairs.

## 2. Materials and methods

### 2.1. Array measurement

#### 2.1.1. Experimental setup

**Fig. 3.**
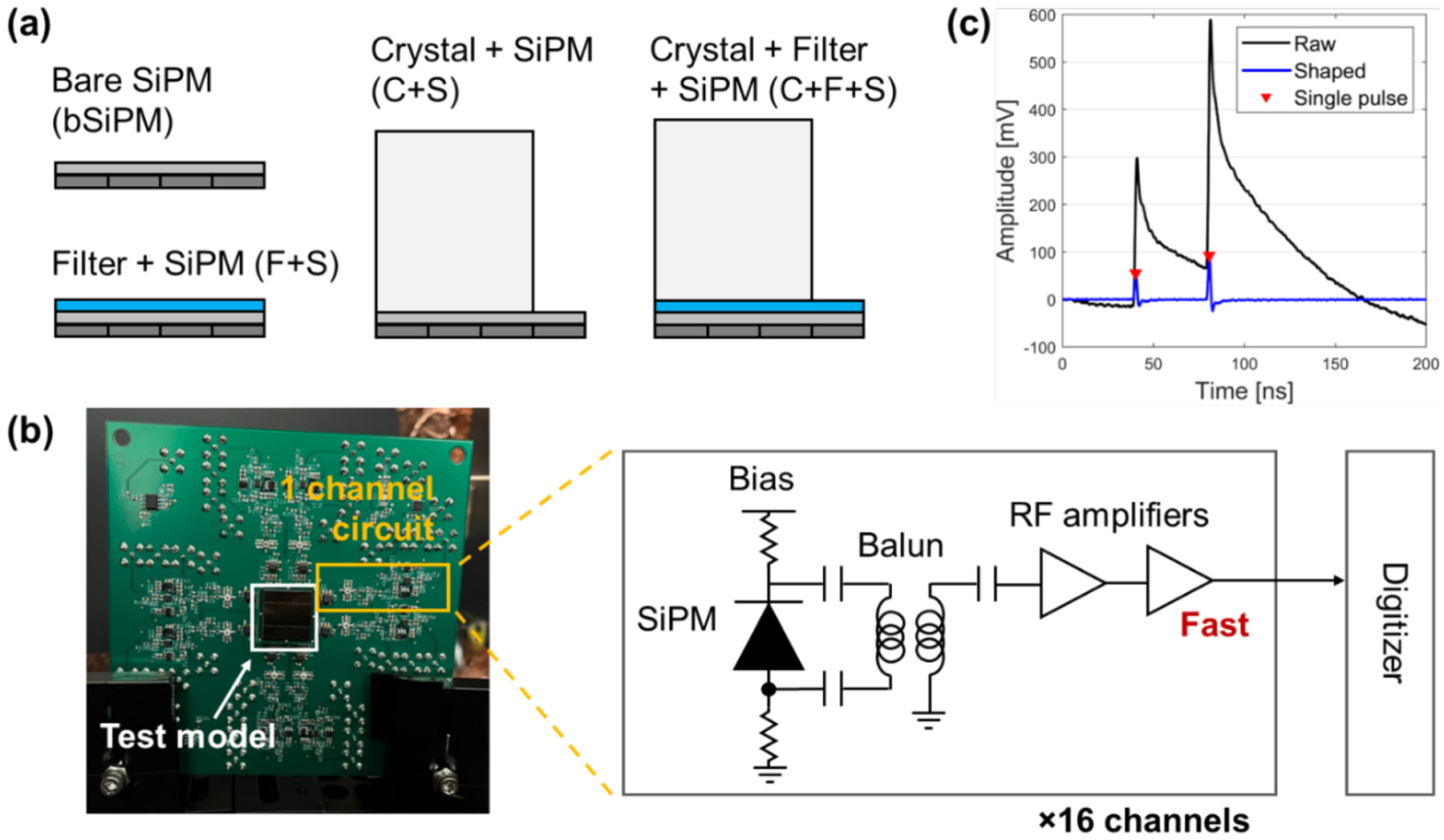
Array SiPM measurement setup. (a) Schematics of the tested configurations. bSiPM, F + S, C + S, and C + F + S denote bare SiPM, filter + SiPM, crystal + SiPM, and crystal + filter + SiPM, respectively. (b) 16-channel SiPM readout board shown with the schematic of the circuit. (c) Example waveform of pulse train sampled by the digitizer. The single pulses (red points) were counted from the shaped pulse train (blue).

Fig. 3(a) shows four different detector configurations for evaluation: bare SiPM (bSiPM), filter-coupled SiPM (F + S), a monolithic LYSO crystal-coupled SiPM (C + S), and LYSO coupled to the filter-coupled SiPM (C + F + S). We tested a 16 × 16 mm^2^ SiPM array (AFBRS4N44P164 M; Broadcom) consisting of 4 × 4 channels in 4-mm pitch with a single channel size of 3.84 × 3.74 mm^2^. The sensor array shares a continuous epoxy window. We selected BG40 (Schott) as an optical bandpass filter (also suggested by Ref. [19]) that features high transparency in the range of 350–630 nm (Fig. 2), as a filter for crosstalk photons. The size of the filter was 16 × 16 × 1 mm^3^ covering the entire SiPM array without any reflector wrapped. A polished 12 × 12 × 20 mm^3^ LYSO (Epic Crystal) was wrapped with Teflon tape and coupled over a 3 × 3 section of channels in the SiPM array. Therefore, 9 channels with LYSO covered were analyzed and reported throughout this work. An optical grease (BC630 with a refractive index of 1.465, Saint-Gobain) was used as a coupling medium between detector components.

To read out the SiPM signals, we employed a sixteen-channel front-end circuit board (Fig. 3(b)). Each channel consisted of a balun transformer and two-stage radio-frequency amplifier cascade to produce fast single photon pulse with good signal-to-noise-ratio, i.e., beneficial for single photon counting of optical photons. The details of the electronics chain for each channel of readouts are described in Ref. [25]. Waveforms of the fast pulses were digitized by a DRS4-based multi-channel digitizer (CAEN V1742) with a sampling rate of 5 GSa/s.

In the absence of a radioactive source, the SiPM was biased with overvoltage (OV from 7 V to 12 V inside a light-tight box. A dry nitrogen flush, maintained at 20 °C, was blown over the detector array to maintain room temperature operation. A software trigger was used to randomly capture SiPM noise events within a 200 ns capture window. We discarded the pulses cut off by the dynamic range of the digitizer (1 V) to discriminate events involving intrinsic Lu-176 decay. Digital pulses were processed with a 350–550 MHz bandpass filter with baseline restoration, yielding sharp single-photon pulses. Single photon pulses were identified using a peak-finding algorithm on digitized traces (Fig. 3 (c)), where the minimum temporal distance of the separated pulses was 1 ns.

#### 2.1.2. Noise rate measurement

Noise pulses were counted by identifying single pulse peaks (indicated in red in Fig. 3(c)) of every 200-ns capture regardless of pulse amplitude. Digitized Geiger cell discharges (single- and few-photon equivalent level voltage signals) in these capture windows are the result of dark counts, crosstalk, and after-pulsing, constituting a “baseline” of noise for each given configuration (i.e., not the product of luminescence from the interaction of ionizing radiation in the scintillator). We encompass all these event types that could be falsely recognized as scintillation photons with the term “noise”. Approximately 30,000 captures were analyzed for each configuration and OV. Noise count histograms were generated and fitted with Poisson distributions to assess the mean noise count for each channel. Noise rate (NR) was defined as the mean noise count per second generated by each SiPM channel. Note that NR is not related to electronic noise in the experimental setup.

#### 2.1.3. Optical photon pileup probability measurement

From the histogram of the single pulse peak amplitude, a voltage pulse with an amplitude higher than a single photon equivalent amplitude was considered a pileup event. For single channel measurements (Section 2.2.2), we regarded pileup as identical to crosstalk because the origin of the crosstalk was only the single channel itself and the NR was relatively low. In these array measurements, however, optical photon pileup is less probable because the correlation between the primary noise and crosstalk can be lost by inter-channel crosstalk sharing and external propagation. Therefore, in this section, we refer to the probability of one or more pileups being detected for each event as a pileup probability, rather than a crosstalk probability.

### 2.2. Single channel measurement

#### 2.2.1. Experimental setup

**Fig. 4.**
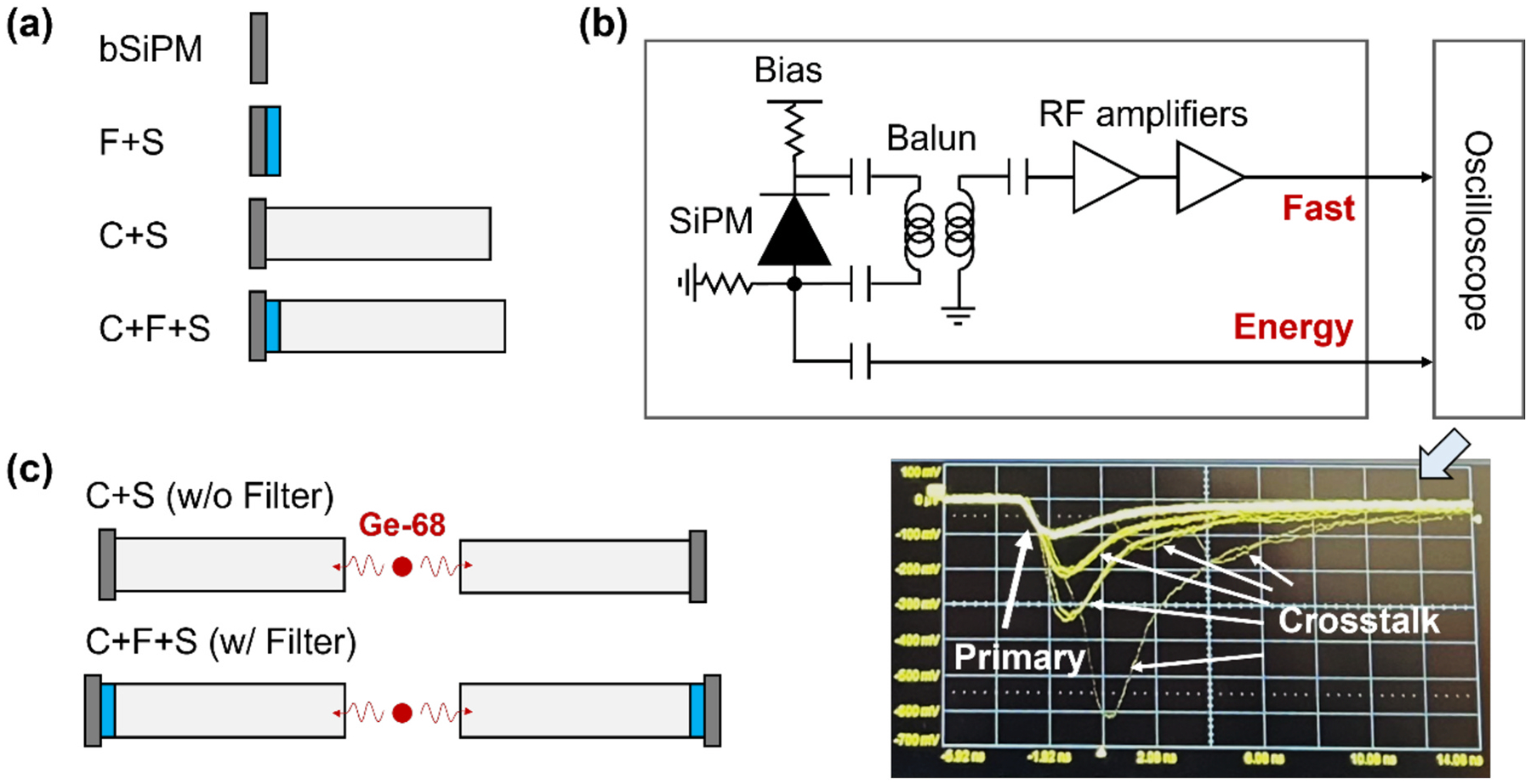
Single channel SiPM measurement setup. (a) Schematics of the configurations for noise characterization: bSiPM, F + S, C + S, and C + F + S denote bare SiPM, filter + SiPM, crystal + SiPM, and crystal + filter + SiPM, respectively. (b) Schematic of the single channel SiPM readout circuit shown with the example of noise waveforms on the oscilloscope. (c) Coincidence setup for light output and coincidence time resolution measurement.

The SiPM was a single pixel of the same device used for array measurements, described in Section 2.1 (AFBR-S4N44P014 M). We used an LGSO crystal (Fast-LGSO; Oxide) with a size of 3 × 3 × 20 mm^3^ and mechanically polished surfaces. The tested configurations were similar to those of Section 2.1.1 (Fig. 4(a)). A 3 × 3 × 1 mm^3^ BG40 filter was used for F + S. In the case of C + F + S, a 3 × 3 × 1 mm^3^ BG40 filter was coupled to the LGSO with Meltmount (n = 1.582, Cargille) and was wrapped with Teflon, along with the crystal. All other interfaces were coupled with optical grease.

The SiPM signal passed through a single-channel readout board with a circuit design similar to that used in Section 2.1.1 (Fig. 4(b)). The pulses were sampled by a digital oscilloscope with a sampling rate of 40 GSa/s, applying OV from 10 V to 16 V for every configuration.

#### 2.2.2. Crosstalk characterization

A threshold at 20% of the single photon pulse amplitude was applied with the hardware triggering of the oscilloscope. The methodology of extracting the crosstalk was similar to that of [19]. A representative template of a 20-ns single photon pulse shape was created by averaging waveforms with the amplitude within a narrow window of ±5% around the mean single photon pulse amplitude. The crosstalk photons were extracted by subtracting the aligned template from the captured 20-ns pulse train; thus, the crosstalk pulses appeared with the primary noise pulse eliminated, while only the baseline with electronic noise was left in the case of a crosstalk-free event. Applying the same leading-edge threshold at half of the single photon pulse amplitude, the time delay of the crosstalk photon detection (timestamp of the template-subtracted pulse) relative to the primary dark noise (timestamp of the original pulse) was measured event-by-event.

The number of pileup crosstalk photons for each pulse was determined by segmenting the distribution of the pulse amplitudes. For instance, Fig. 7(a) shows clear separations between the adjacent peaks with <5% relative widths, where each peak represents the number of pileups. We applied a 2 ns window to the time delay, assuming that the photons detected later than 2 ns after the primary noise was another uncorrelated primary noise. Still, the assumption is not perfect because some delayed or external crosstalk would undergo delays of several nanoseconds [3]. The probability as a function of the number of crosstalk photons detected, which was referred to as crosstalk probability, was assessed from the resulting delay-amplitude distribution.

#### 2.2.3. Noise rate

NR of the single channel was measured using a method similar to that described in Sections 2.1.1 and 2.1.2. Random capturing of pulses was performed from a “line” trigger on the oscilloscope. We counted the number of noise pulses within a 200-ns capture using a peak finding algorithm to calculate the NR of each configuration. The peak finding parameters were set to regard subsequent pulses later than 0.6 ns from the primary pulse as new separate pulses.

#### 2.2.4. Setup for light output and coincidence time resolution measurements

To investigate the impact of the filter on the timing performance of the detectors, we used a coincidence measurement setup described in Fig. 4(c) with increasing OV from 10 V to 20 V. Pairs of models C + S (without filter) and C + F + S (with filter) described in Section 2.2.1 were tested with the same single-channel high-frequency readout board and digital oscilloscope. We placed a Ge-68 point source between the detectors to acquire 511-keV annihilation events by triggering coincidence events, determined from the energy channels of both detectors.

From the energy pulse amplitude spectrum, the mean pulse amplitude at 511-keV photopeak was assessed to compare the relative light output with and without coupling the filter. CTR was defined as a full width at half-maximum (FWHM) of the time delay histogram between the two detectors. Time pickoff was performed with a simple leading-edge discriminator (i.e., no digital baseline selection or corrections were performed). For every optical bandpass configuration and OV, we report CTR at the optimum leading-edge threshold.

## 3. Results

### 3.1. Array measurement

#### 3.1.1. Noise rate

**Fig. 5.**
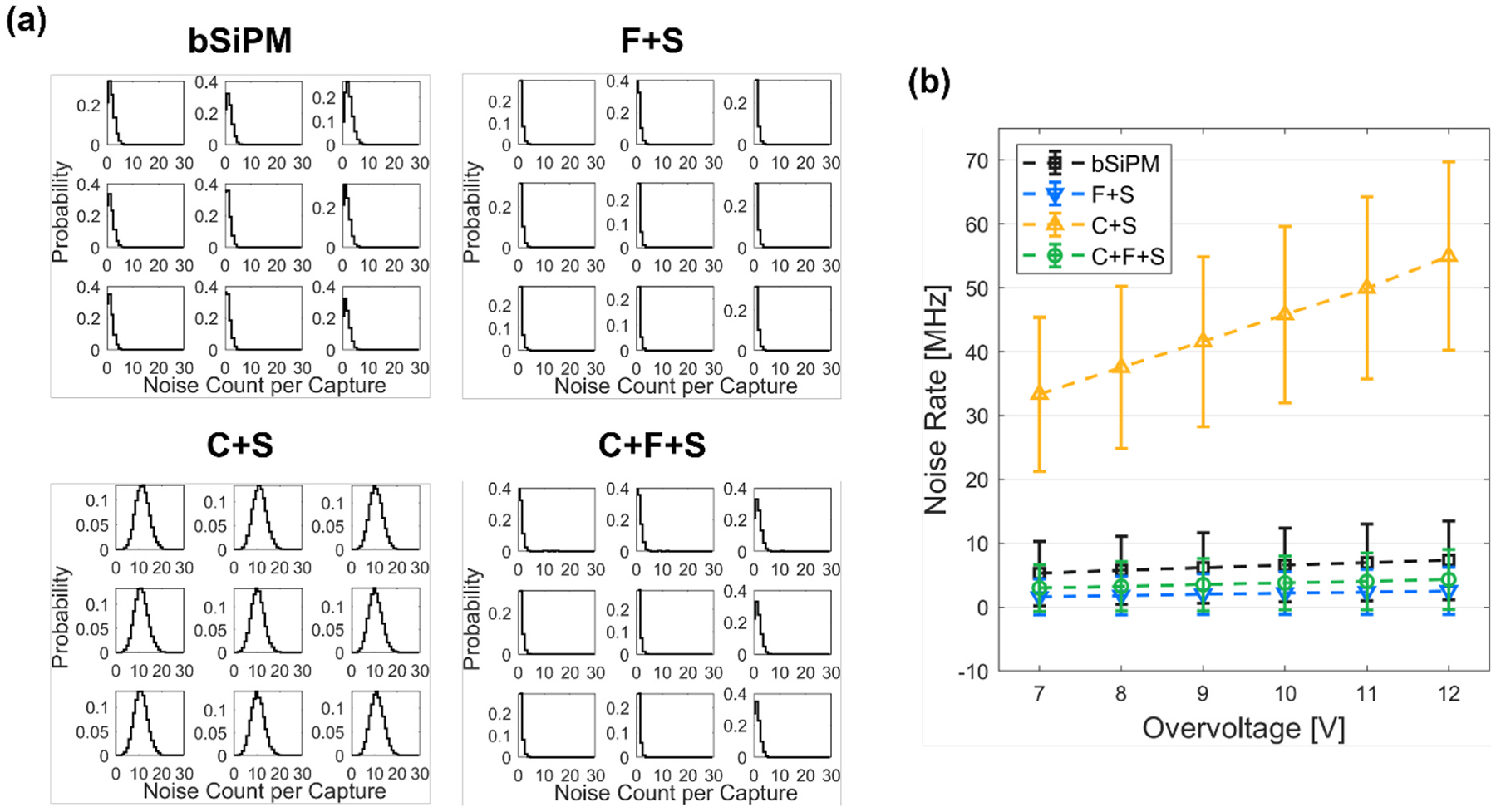
Noise rate measured of the configurations with the array SiPM. (a) Noise count distributions of 3 × 3 channels from randomly captured 200-ns waveforms at OV = 12 V. (b) Noise rates of the tested configurations with sweeping the overvoltage.

Per-channel noise count distributions of four different configurations at OV = 12 V are shown in Fig. 5(a). For C + S, 11 noise pulses on average were captured within a 200-ns window, which was 7 times higher than that for the bare SiPM, demonstrating the noise amplification that external optical crosstalk can produce for crystal-coupled SiPMs. Based on these distributions, we calculated the NR of every configuration as a function of OV (Fig. 5(b)). The data points and the error bars indicate the mean and standard deviation derived from Poisson fits to the distributions, averaged over nine channels. A remarkably high NR of C + S indicates that coupling a monolithic crystal directly to the SiPM resulted in a large external crosstalk photon sharing throughout the SiPM channels. However, as shown in the case of C + F + S, coupling the filter suppressed the NR by a factor of 12 compared to C + S for overall OVs (4.3 MHz versus 55 MHz at OV = 12 V). The NR of F + S was lower than that of bSiPM (2.5 MHz versus 7.3 MHz at OV = 12 V), implying that a non-negligible amount of crosstalk photons undergoes total internal reflection at the window surface of bSiPM, which were absorbed in the filter in the F + S configuration.

#### 3.1.2. Pileup probability

**Fig. 6.**
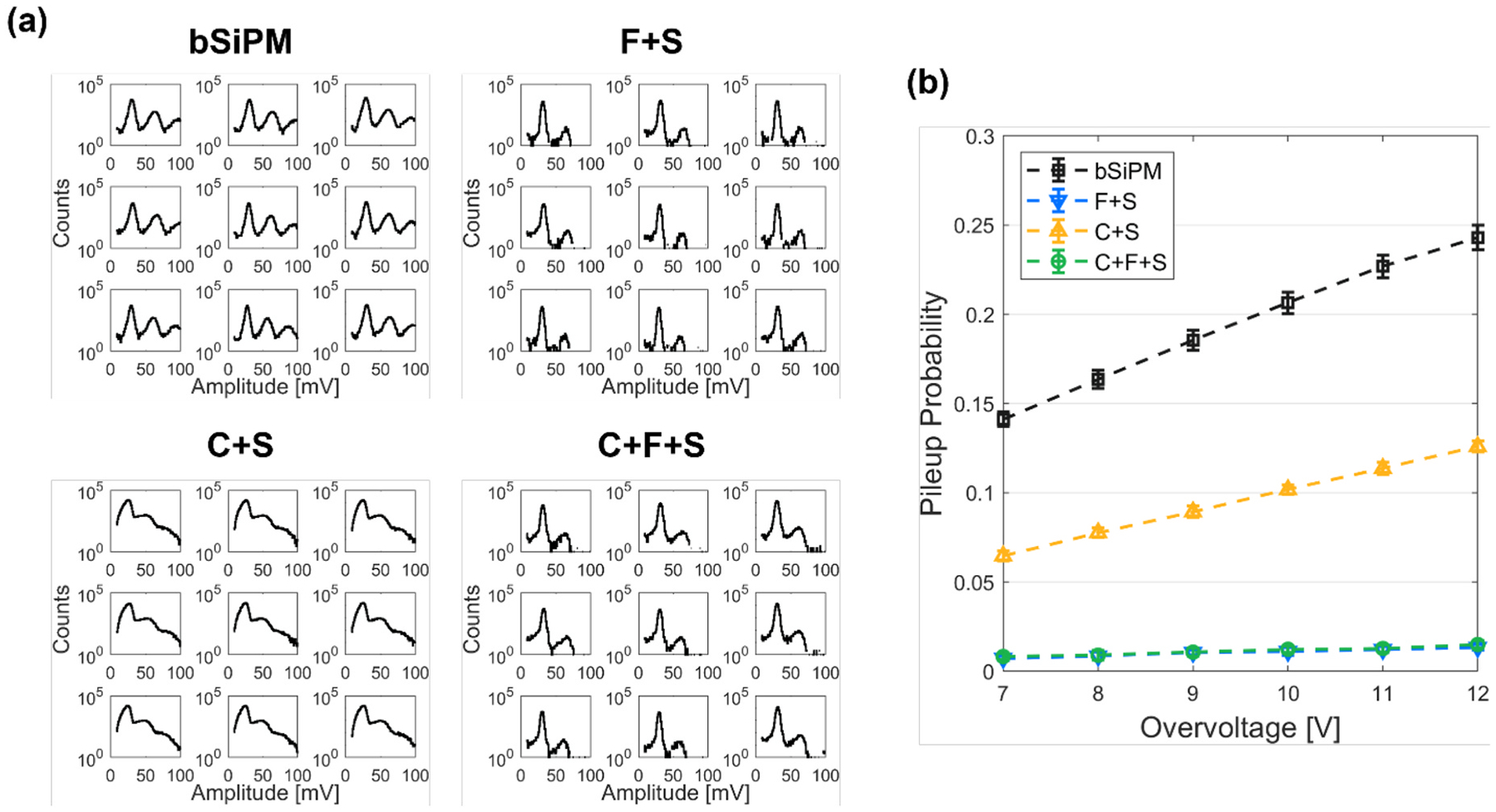
Pileup probability of the configurations with the array SiPM. (a) Single noise pulse amplitude distributions of 3 × 3 channels from randomly captured 200-ns waveforms at OV = 12 V. (b) Pileup probabilities of the tested configurations with sweeping the overvoltage.

Fig. 6(a) shows the histograms of the single pulse amplitudes at OV = 12 V for each channel. Unlike the other configurations, C + S shows blurred peaks, which is due to high NR in combination with undershoot produced from pulse shaping in the pulse trains (see pulse undershoot in Fig. 3(c)). When the filter was coupled, i.e., F + S and C + F + S, pulses with more than one pileup were significantly reduced.

The probability of one or more pileup events was assessed based on the amplitude histogram (Fig. 6(b)). The pileup probabilities of the F + S and C + F + S were comparable, implying that the effect of the pileup due to external optical crosstalk through the crystal is negligible when the filter is used. Interestingly, a significant reduction of pileup for C + S, as compared to the bSiPM configuration, was observed. This also supports the explanation in Section 3.1.1. Intra-channel crosstalk photons can pile up on the primary noise due to reflection at the window-air interface for a bSiPM configuration, while the same photons would likely enter the crystal in the case of C + S, because the refractive index of the crystal (1.82) is higher than that of the epoxy window (~1.5). These photons would appear as separate noise pulses with either spatial or temporal correlations lost, thus resulting in increased NR, which was observed in Fig. 5(a). Similarly, the match of refractive indices between the filter (1.53) and the window allowed a large portion of crosstalk photons to propagate through the filter where they can be absorbed, resulting in a considerable reduction in NR for F + S.

### 3.2. Single channel measurement

#### 3.2.1. Crosstalk characterization

**Fig. 7.**
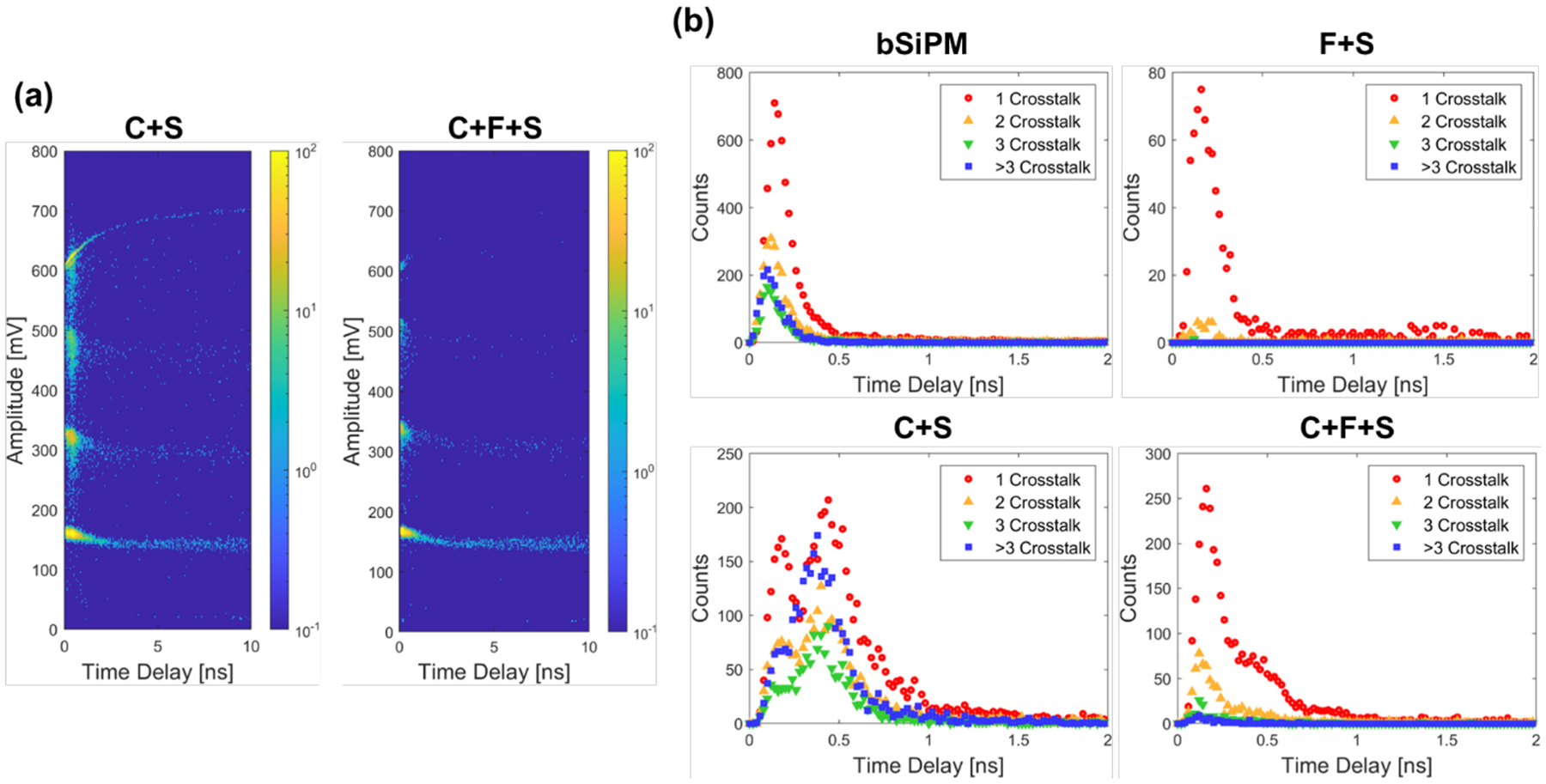
(a) Delay-amplitude and (b) delay histograms of crosstalk photons in the single channel measurements at OV = 16 V. (a) Was used for classifying the events into the number of pileups and obtaining (b) by projecting to the time delay axis.

The delay-amplitude distributions of C + S and C + F + S at OV = 16 V are plotted in Fig. 7(a). The pulse amplitudes were discretized horizontally by the number of pileups, where the crosstalk is a dominant source for single-channel measurements. Fig. 7(b) shows the distributions of the crosstalk photon time delay relative to the primary discharge, which is also the projection of Fig. 7(a) to the time delay axis. Crosstalk photons for the bSiPM configuration were detected within 0.5 ns, as well as F + S. An additional peak was observed in C + S at 0.4 ns regardless of the number of crosstalk, which resulted from external crosstalk photons that arrived at the SiPM after propagating through the scintillation crystal element. By applying the filter, this peak was largely suppressed, which is another representation of the impact of the filter on the external crosstalk.

**Fig. 8.**
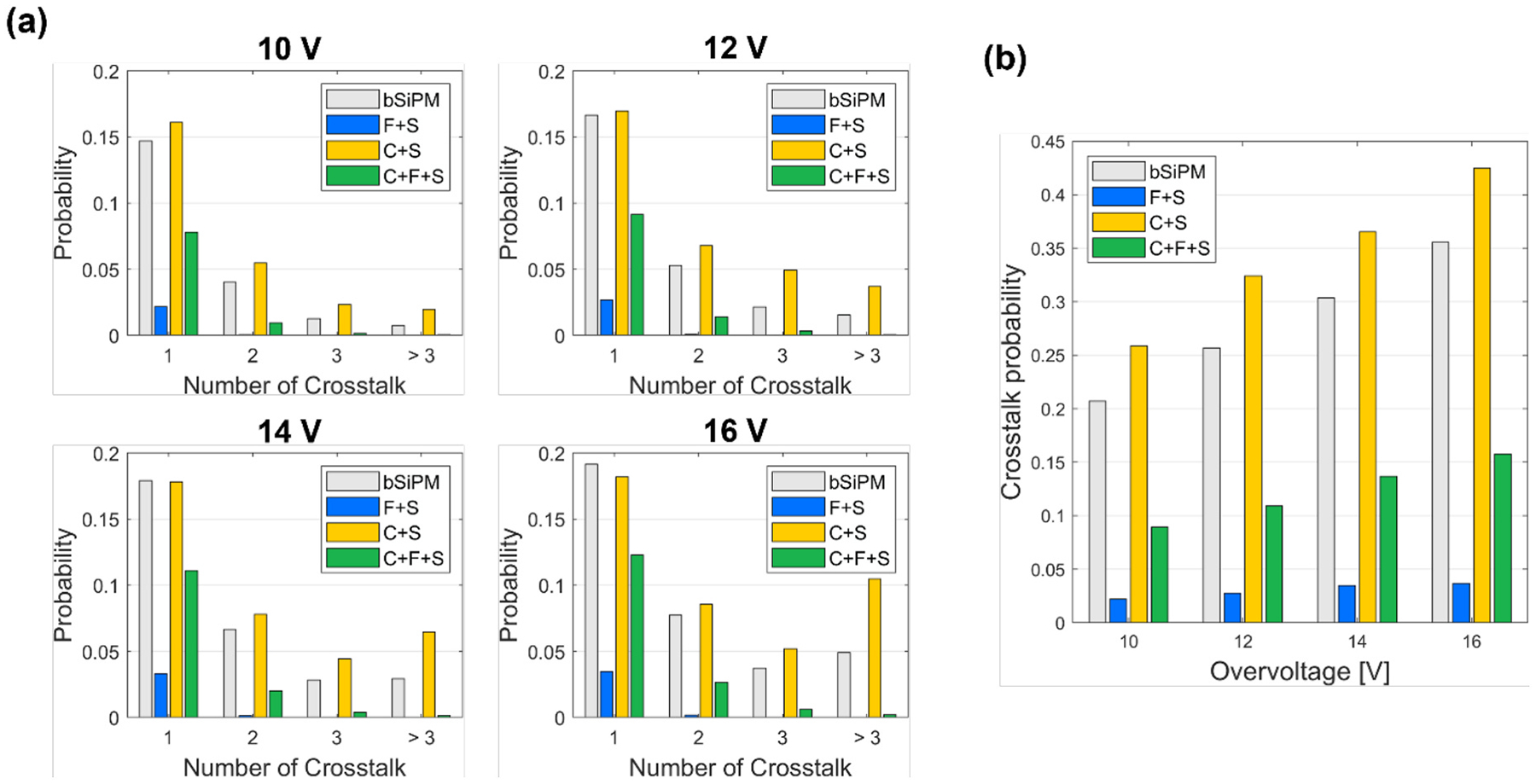
Crosstalk probabilities of the single channel configurations (a) respective to the number of involved crosstalk and (b) summed over the numbers of crosstalk.

Fig. 8(a) shows the probability of a respective number of detected crosstalk photons per primary discharge (due to a dark count in these measurements). With increasing OV, the growth of the probability of three or more crosstalk was significant for C + S, while it was suppressed for C + F + S. In the case of F + S, the probability of detecting one crosstalk photon was approximately a few percent, and <1% probability for two or more crosstalk. Fig. 8(b) shows the summation of the probabilities over the number of detected crosstalk photons, which corresponds to the probability of detecting one or more crosstalk photons for all OV values. C + F + S exhibited approximately 64% less crosstalk probability compared to C + S for all OV values due to the impact of the filter. The increment in the crosstalk probability of C + S compared to the bSiPM configuration, which corresponds to the contribution of the external crosstalk caused by the crystal coupling, ranged from 5.2% (12 V) to 6.9% (16 V). The crosstalk probability of the bare SiPM was 26% at OV = 12 V, which was comparable to that reported on the datasheet of the SiPM (23% at 25 °C) [22].

#### 3.2.2. Noise rate

**Fig. 9.**
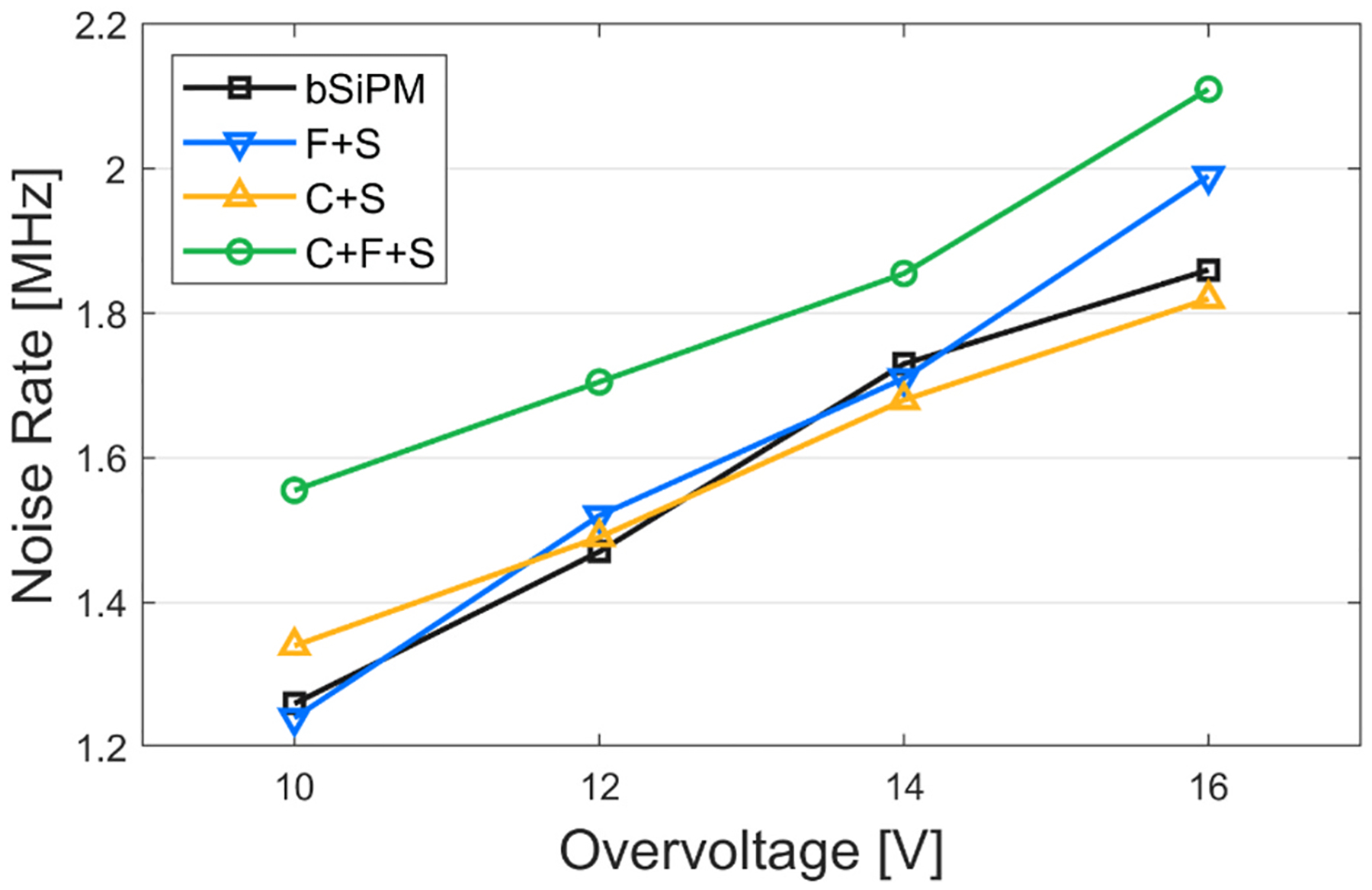
False trigger rate of the single channel configurations depending on overvoltage.

Unlike the array SiPM measurements in Section 3.1.1, all single-channel configurations exhibited comparable NRs. (Fig. 9). This is somewhat predictable, as the major source of the primary dark noise in these configurations is thermionic emission in the SiPM microcells regardless of filter coupling. In the single channel setup where the origin and the receiver of the crosstalk are the same, most of the crosstalk was detected within 1 ns from the primary discharge (Fig. 7(b)) without triggering additional single pulses. Therefore, it is reasonable to assume the NR of the single-channel SiPM is primarily characteristic of the dark count rate. The NR values (1.5–1.7 MHz at 20 °C) were in good agreement with the dark count rate reported by the manufacturer on the datasheet (1.7 MHz at 25 °C) at OV = 12 V [22]. High per-channel NR in the bSiPM array measurement (7.3 MHz at OV = 12 V), as compared to that of single-channel bSiPM, suggests that the crosstalk photon propagation between the channels through the SiPM window can be significant.

#### 3.2.3. Light output and coincidence time resolution

**Fig. 10.**
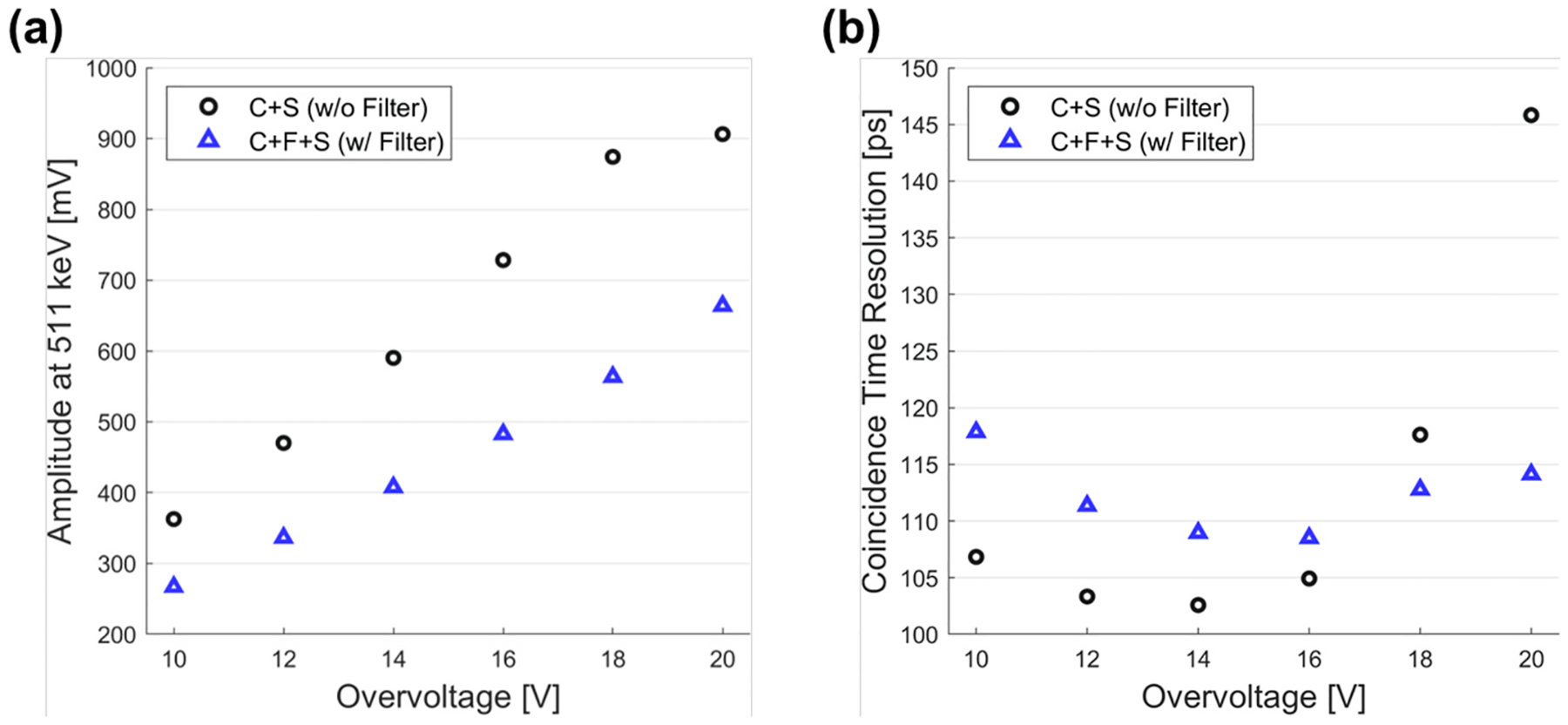
Coincidence measurement results. (a) The mean amplitude of the 511-keV scintillation signals. (b) Coincidence time resolutions of the configurations C + S and C + F + S depending on overvoltage.

Comparing the light output in Fig. 10(a), coupling the filter resulted in a 25% and 35% decrease in the 511-keV pulse amplitudes at OV = 10 V and 16 V, respectively. Given that only approximately 3% of Fast LGSO’s scintillation photons are absorbed by the 1-mm thick BG40 filter (Fig. 2), this pulse amplitude reduction is primarily due to filtered crosstalk photons. Weighting the crosstalk with respective probability reported in Section 3.2.1 and Fig. 8(a), the expected number of detected crosstalk photons per avalanche in the microcell are 0.93 and 0.20 for C + S and C + F + S at OV = 16 V, respectively. Thus, the expected fraction of signal amplitude remaining with the filter in place is 1.20/1.93 or 62%. This is in general agreement with the 35% reduction of 511-keV pulse amplitude due to filter coupling, explaining the pulse amplitude decrease with the optical filter in place (i.e., primarily a loss of noise and not signal).

CTR for the single pixel detector pairs is shown in Fig. 10(b). C + S and C + F + S achieved best CTRs of 103 ± 1.0 ps and 109 ± 1.2 ps at OV = 14 V and 16 V, respectively. Despite the 64% reduction of crosstalk, C + F + S showed degraded CTR. This degradation could potentially result from a minor increase in scintillation photon transit time jitter and a minor decrease in light collection efficiency, due to the additional optical interfaces (crystal-filter and filter-SiPM). In addition, considering the CTR proportionality to 1/√(N) where the N denotes the number of scintillations, the expected CTR if the signal amplitude loss was entirely due to scintillation photon statistics is 103/√(1.20/1.93) = 131 ps, whereas the C + F + S configuration showed significantly better CTR. This further supports the assertion that the loss in signal amplitude observed between the C + S and C + F + S configurations is primarily due to the absorption of optical crosstalk photons and not scintillation photons.

It is also noteworthy that when OV higher than 16 V was applied, C + F + S showed only a ~5 ps worse CTR, as compared to the optimal CTR. In contrast, C + S showed a rapid degradation by further increasing OV. This suggests that the filter enables stable operation of the detectors at very high OV.

## 4. Discussion

Overall, the presented results demonstrate that substantial suppression of external crosstalk is possible by including an optical bandpass filter between scintillators and SiPMs. The filter could reduce both the NR and pileup probability of array C + S towards the levels comparable to those of F + S (Figs. 5(b) and 6(b)). The NR of the array C + F + S was 4.3 MHz at OV = 12 V, which was 92% less than that observed in the C + S configuration. The results suggest that using an off-the-shelf optical bandpass filter is a relatively straightforward way to significantly reduce external optical crosstalk in scintillation detectors that employ SiPM arrays, operated at high OV. The single-channel measurements also showed a significant reduction in crosstalk probability (Fig. 8(a)) along with suppressed external crosstalk (Fig. 8(b)). A small degradation in CTR due to the filter was observed, but the filter-coupled detector configuration outperformed that case with no filter at high OV (Fig. 10).

By comparing the characterized noise properties of different sensor/filter configurations, external crosstalk was comprehensively studied in a single SiPM and an array of SiPMs. The NR comparison between array bSiPM and array F + S shows a significant inter-channel sharing of crosstalk photons through total internal reflection due to a large difference in refractive indices (~1.5 versus 1) for the SiPM epoxy window and air, respectively. Coupling a large-volume crystal to the SiPM array surface, on the other hand, enables external crosstalk photons to enter the crystal and arrive at other channels in the SiPM array after propagating through the crystal. Therefore, these crosstalk photons appeared as separate noise pulses rather than pileup events, resulting in a significant increase in NR and reduced crosstalk probability for array C + S compared to bSiPM. A similar effect of the filter and crystal was observed from crosstalk probability in single-channel measurements, while comparable NRs of single-channel configurations indicate that microcell firing due to dark noise was irrelevant to either the filter or crystal coupling.

Coupling an optical bandpass filter could be exploited in large-area segmented and monolithic crystal-based scintillation detectors to significantly reduce external crosstalk. For the 1-to-1 combination of a 3 × 3 × 20 mm^3^ crystal and a single-channel SiPM, the filter did not show an obvious advantage in terms of CTR because of increased scintillation photon transit time jitter in the setup used in this study. However, a better CTR of C + F + S than that of C + S with 1/√(*N*) proportionality applied suggests that the filter offers improvement in CTR through crosstalk reduction. Moreover, external crosstalk is amplified by the presence of a crystal that serves as a reflector for crosstalk photons toward the SiPM, resulting in CTR divergence at low bias voltage [17]. This also explains the suppressed degradation of CTR at OV > 16 V by coupling the filter (Fig. 10(b)). When implemented in a large area detector employing an SiPM array, the benefit of the filter is emphasized by allowing the operation at sufficiently high bias voltage to yield good CTR at room temperature. For instance, a detector consisting of a highly pixelated crystal array and channel-multiplexed readout scheme can benefit from crosstalk filtering, potentially improving the crystal resolving capability and time resolution. Another case is monolithic crystal-based detectors where the external crosstalk can freely spread throughout the detector array, which can negatively impact estimates for position and time of interaction. Besides, other various SiPM applications can benefit from the employment of the optical filter, such as its potential use as a light guide in light-sharing readout of segmented arrays of scintillation crystals. The improvement is expected to be significant for applications employing scintillators with low light yield, short wavelength emission, and substantial light sharing across the entire volume.

We experimentally quantified the impact of a BG40 filter on the noise characteristics of the LGSO/LYSO scintillation crystal-based detectors coupled to SiPMs. In the absence of scintillation crystals, the results were in good agreement with those of [19], while we have taken the study of employing an optical bandpass with SiPMs a step further by quantifying the external optical crosstalk reduction when scintillation crystal elements are coupled, a case which further exacerbates the issue of external crosstalk. The contribution of an optically coupled crystal to crosstalk was characterized by comparing the bSiPM and C + S in NRs of array models and time delay distributions of single models. The NR, crosstalk probability, and crosstalk time delay characterized in this study can be easily integrated into the realistic modeling of the detectors using SiPMs. Depending on the emission spectra of the scintillator and crosstalk, a selection of various bandpass windows can be made from commercially available optical filters. This work focused on the combination of a Schott BG40 filter and LGSO/LYSO crystals with peak emission wavelengths of ~420 nm, thus a similar behavior is expected for other crystals with similar emission spectrum such as LSO.

## 5. Conclusion

The use of an optical bandpass filter demonstrated a significant reduction in external crosstalk for single SiPM and a SiPM array coupled to scintillation crystal elements wrapped in high-efficiency optical reflectors, as evidenced by substantially reduced noise rate of the array SiPM and the crosstalk probability of the single SiPM. By employing a 1-mm thick BG40 filter, a 92% reduction in noise rate and a 64% reduction in crosstalk probability were achieved for array and single SiPM detectors, respectively. This technique could have a substantial impact on large-area detector modules employing segmented or monolithic scintillation crystal volumes coupled to SiPMs, where the reduction in external optical crosstalk can enable higher operating voltage at room temperature.

## Data Availability

Data will be made available on request.

## References

[1] Gundacker S, Heering A, The silicon photomultiplier: fundamentals and applications of a modern solid-state photon detector, Phys. Med. Biol 65 (2020) 17TR01, 10.1088/1361-6560/ab7b2d. 32109891

[2] Ota R, Photon counting detectors and their applications ranging from particle physics experiments to environmental radiation monitoring and medical imaging, Radiol. Phys. Technol 14 (2021) 134–148, 10.1007/s12194-021-00615-5. 33742329

[3] Acerbi F, Gundacker S, Understanding and simulating SiPMs, Nucl. Instrum. Methods Phys. Res 926 (2019) 16–35, 10.1016/J.NIMA.2018.11.118.

[4] Simon F, Silicon photomultipliers in particle and nuclear physics, Nucl. Instrum. Methods Phys. Res 926 (2019) 85–100, 10.1016/j.nima.2018.11.042.

[5] Garutti E, Silicon photomultipliers for high energy physics detectors, J. Instrum 6 (2011) C10003, 10.1088/1748-0221/6/10/C10003.

[6] Park H, Yi M, Lee JS, Silicon photomultiplier signal readout and multiplexing techniques for positron emission tomography: a review, Biomed Eng Lett 12 (2022) 263–283, 10.1007/s13534-022-00234-y. 35892029 PMC9308856

[7] Roncali E, Cherry SR, Application of silicon photomultipliers to positron emission tomography, Ann. Biomed. Eng 39 (2011) 1358–1377, 10.1007/s10439-011-0266-9. 21321792 PMC3069330

[8] Cates JW, Steele J, Balajthy J, Negut V, Hausladen P, Ziock K, Brubaker E, Front-end design for SiPM-based monolithic neutron double scatter imagers, Sensors 22 (2022) 3553, 10.3390/s22093553. 35591242 PMC9101142

[9] Morishita Y, Ye Y, Mata L, Pozzi SA, Kearfott KJ, Radon measurements with a compact, organic-scintillator-based alpha/beta spectrometer, Radiat. Meas 137 (2020) 106428, 10.1016/j.radmeas.2020.106428.

[10] Kataoka J, Kishimoto A, Nishiyama T, Fujita T, Takeuchi K, Kato T, Nakamori T, Ohsuka S, Nakamura S, Hirayanagi M, Adachi S, Uchiyama T, Yamamoto K, Handy Compton camera using 3D position-sensitive scintillators coupled with large-area monolithic MPPC arrays, Nucl. Instrum. Methods Phys. Res 732 (2013) 403–407, 10.1016/j.nima.2013.07.018.

[11] Jiang J, Shimazoe K, Nakamura Y, Takahashi H, Shikaze Y, Nishizawa Y, Yoshida M, Sanada Y, Torii T, Yoshino M, Ito S, Endo T, Tsutsumi K, Kato S, Sato H, Usuki Y, Kurosawa S, Kamada K, Yoshikawa A, A prototype of aerial radiation monitoring system using an unmanned helicopter mounting a GAGG scintillator Compton camera, J. Nucl. Sci. Technol 53 (2016) 1067–1075, 10.1080/00223131.2015.1089796.

[12] Villa F, Severini F, Madonini F, Zappa F, Spads and sipms arrays for long-range high-speed light detection and ranging (Lidar), Sensors 21 (2021) 3839, 10.3390/s21113839. 34206130 PMC8199503

[13] Rando R, Corti D, Dazzi F, De Angelis A, Dettlaff A, Dorner D, Fink D, Fouque N, Grundner F, Haberer W, Hahn A, Hermel R, Korpar S, Mezek GK, Maier R, Manea C, Mariotti M, Mazin D, Mehrez F, Mirzoyan R, Podkladkin S, Reichardt I, Rhode W, Rosier S, Schultz C, Stella C, Teshima M, Wetteskind H, Zavrtanik M, Silicon Photomultiplier Research and Development Studies for the Large Size Telescope of the Cherenkov Telescope Array, 2015. http://arxiv.org/abs/1508.07120.

[14] Miyamoto H, Teshima M, SiPM development for the imaging Cherenkov and fluorescence telescopes, Nucl. Instrum. Methods Phys. Res 623 (2010) 198–200, 10.1016/j.nima.2010.02.194.

[15] Dalla Mora A, Di Sieno L, Behera A, Taroni P, Contini D, Torricelli A, Pifferi A, The SiPM revolution in time-domain diffuse optics, Nucl Instrum Methods Phys Res A 978 (2020) 164411, 10.1016/j.nima.2020.164411.

[16] Otte AN, Garcia D, Nguyen T, Purushotham D, Characterization of three high efficiency and blue sensitive silicon photomultipliers, Nucl. Instrum. Methods Phys. Res 846 (2017) 106–125, 10.1016/j.nima.2016.09.053.

[17] Gola A, Ferri A, Tarolli A, Zorzi N, Piemonte C, SiPM optical crosstalk amplification due to scintillator crystal: effects on timing performance, Phys. Med. Biol 59 (2014) 3615–3635, 10.1088/0031-9155/59/13/3615. 24922188

[18] Merzi S, Brunner SE, Gola A, Inglese A, Mazzi A, Paternoster G, Penna M, Piemonte C, Ruzzarin M, NUV-HD SiPMs with metal-filled trenches, J. Instrum 18 (2023) P05040, 10.1088/1748-0221/18/05/P05040.

[19] Masuda T, Ang DG, Hutzler NR, Meisenhelder C, Sasao N, Uetake S, Wu X, Demille D, Gabrielse G, Doyle JM, Yoshimura K, Suppression of the optical crosstalk in a multi-channel silicon photomultiplier array, Opt. Express 29 (2021) 16914, 10.1364/OE.424460. 34154244

[20] Barton P, Stapels C, Johnson E, Christian J, Moses WW, Janecek M, Wehe D, Effect of SSPM surface coating on light collection efficiency and optical crosstalk for scintillation detection, Nucl. Instrum. Methods Phys. Res 610 (2009) 393–396, 10.1016/j.nima.2009.05.187.

[21] Nepomuk Otte A, On the efficiency of photon emission during electrical breakdown in silicon, Nucl. Instrum. Methods Phys. Res 610 (2009) 105–109, 10.1016/j.nima.2009.05.085.

[22] Broadcom, AFBR-S4N44P164M: 4×4 NUV-MT Silicon Photomultiplier Array Datasheet, 2023.

[23] Gallego L, Rosado J, Blanco F, Arqueros F, Modeling crosstalk in silicon photomultipliers, J. Instrum 8 (2013) P05101, 10.1088/1748-0221/8/05/P05010.

[24] Vinogradov S, Analytical models of probability distribution and excess noise factor of solid state photomultiplier signals with crosstalk, Nucl. Instrum. Methods Phys. Res 695 (2012) 247–251, 10.1016/j.nima.2011.11.086.

[25] Cates JW, Gundacker S, Auffray E, Lecoq P, Levin CS, Improved single photon time resolution for analog SiPMs with front end readout that reduces influence of electronic noise, Phys. Med. Biol 63 (2018) 185022, 10.1088/1361-6560/aadbcd. 30129562

